# A Neuroeconomics Approach to Inferring Utility Functions in Sensorimotor Control

**DOI:** 10.1371/journal.pbio.0020330

**Published:** 2004-09-21

**Authors:** Konrad P Körding, Izumi Fukunaga, Ian S Howard, James N Ingram, Daniel M Wolpert

**Affiliations:** **1**Sobell Department of Motor Neuroscience, Institute of NeurologyUniversity College London, LondonUnited Kingdom

## Abstract

Making choices is a fundamental aspect of human life. For over a century experimental economists have characterized the decisions people make based on the concept of a utility function. This function increases with increasing desirability of the outcome, and people are assumed to make decisions so as to maximize utility. When utility depends on several variables, indifference curves arise that represent outcomes with identical utility that are therefore equally desirable. Whereas in economics utility is studied in terms of goods and services, the sensorimotor system may also have utility functions defining the desirability of various outcomes. Here, we investigate the indifference curves when subjects experience forces of varying magnitude and duration. Using a two-alternative forced-choice paradigm, in which subjects chose between different magnitude–duration profiles, we inferred the indifference curves and the utility function. Such a utility function defines, for example, whether subjects prefer to lift a 4-kg weight for 30 s or a 1-kg weight for a minute. The measured utility function depends nonlinearly on the force magnitude and duration and was remarkably conserved across subjects. This suggests that the utility function, a central concept in economics, may be applicable to the study of sensorimotor control.

## Abstract 2

Economists use the concept of a utility function, which increases with increasing desirability of the outcome, to characterize human decision making. This concept is shown here to apply to the control of movement

## Introduction

In real world situations we often have to choose between possible actions that lead to different outcomes. To provide a computational framework for such a decision process, the notion of a utility function is often used (Neumann and Morgenstern 1944). A utility function assigns to each possible action a number that specifies how desirable each outcome is. In the theory of rational choice, it is assumed that subjects will choose the action that leads to the most desirable outcome and thus the highest utility. The economics literature extensively discusses the problem of having a utility function that depends on two or more variables (Edgeworth 1881; Pareto 1909). For example, people may associate a utility with the number of apples and oranges they are offered. There will be combinations of apples and oranges which have equal utility. Having three apples and three oranges could be judged as being equally good as having ten apples and one orange. These two possibilities would form two points along an “indifference curve” in apple–orange space, representing outcomes with identical utility that are, therefore, equally desirable. Such indifference curves have been extensively studied by economists in terms of goods and services (c.f. Humphrey 1996). The sensorimotor system also has to choose between different actions. The utility of actions will depend on two components—the cost associated with performing an action and the desirability of the outcome. Here we characterize the utility function used by the sensorimotor system by measuring the indifference curves for human subjects experiencing short pulses of force.

In sensorimotor control, utility functions that depend on several variables occur frequently. Consider, for example, unpacking a car after a snowboarding vacation. We could carry all the suitcases at the same time, reducing the time to unpack but maximizing the weight we have to lift concurrently. At the other extreme we could transport each item individually, which would minimize the magnitude of the force required at the expense of a long unpacking duration. The chosen solution is likely to lie somewhere between these two extremes and may reflect an optimal decision based on a utility function that depends on duration and magnitude of the forces. Once a utility function is specified, the decision problem becomes one of solving an optimal control problem, finding the actions that maximize the utility.

A number of studies in the field of optimal sensorimotor control have proposed loss functions (the negative of utility) and derived the optimal actions given these proposed loss functions. For example, the minimum jerk model (Hogan 1984; Flash and Hogan 1985) suggests that people minimize the average squared jerk of the hand (third derivative of position) when making reaching movements. Alternative models have suggested that during reaching people try to minimize the variation of endpoint errors that arise from noise on the motor commands (Harris and Wolpert 1998; Todorov and Jordan 2002). However, these and many other similar studies assume a loss function and compare the predicted behavior with observed behavior, rather than measure the loss function directly. In recent works we have used a statistical approach to infer the loss function, instead of assuming it. We defined the statistics of the errors observed by subjects and showed that they were sensitive to quadratic errors for small errors but that for larger errors they were robust to outliers (Körding and Wolpert 2004). Here we use an alternative approach that is analogous to the approaches used in economics to infer a loss function.

Different movements may be associated with different costs or utility. For example, a utility function could assign a numerical value to each possible movement, characterizing how costly it is to the organism. Here, we have examined how the utility associated with producing a force depends on two parameters, the duration and the magnitude of a force profile (see Materials and Methods for details). The force profiles were smoothed square waves that could be linearly scaled by the duration of the force, *T,* and the maximum value of the force, *F.* On each trial, subjects experienced two force profiles that differed in both *T* and *F.* They then had to choose which of the two force profiles they would experience again. They were told to choose the force that required the least effort. In this two-alternative forced-choice experiment subjects thus indicated their preference for one combination of *F* and *T* over another combination of *F* and *T.* This allowed us to infer indifference curves: Given the choice of two combinations of *F* and *T* that are on the same indifference curve, subjects will have no preference. The associated utility of these force profiles is thus identical. To obtain a full utility function from a set of utility curves we additionally needed to determine the utility of one indifference curve relative to another. This was achieved by finding “doubling points.” A doubling point is a point on one indifference curve that subjects show no preference for when compared to experiencing a point on another indifference curve twice (that is, two smoothed square waves in quick succession—see Materials and Methods for details). Thus we could determine the full utility function.

## Results/Discussion

**Figure 1:**
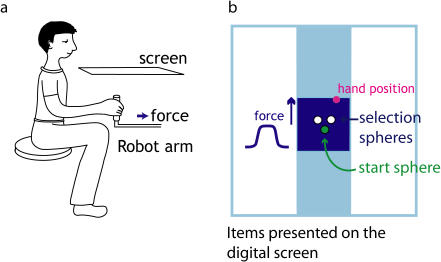
The Experimental Setup The subject's hand position (pink circle) was visible on the screen. The hand movement was restricted to stay within a small area (blue box). The direction of the force is represented by the blue arrow, and the temporal profile of the force is shown by the blue curve.

In a two-alternative forced-choice paradigm, subjects chose which of two experienced force profiles they preferred to experience a second time (see Figure 1). This allowed us to find a set of force profiles to which subjects showed equal preference and were therefore indifferent. Although subjects experience these force profiles as being physically different, they show no preference in terms of which they wish to experience again.

**Figure 2:**
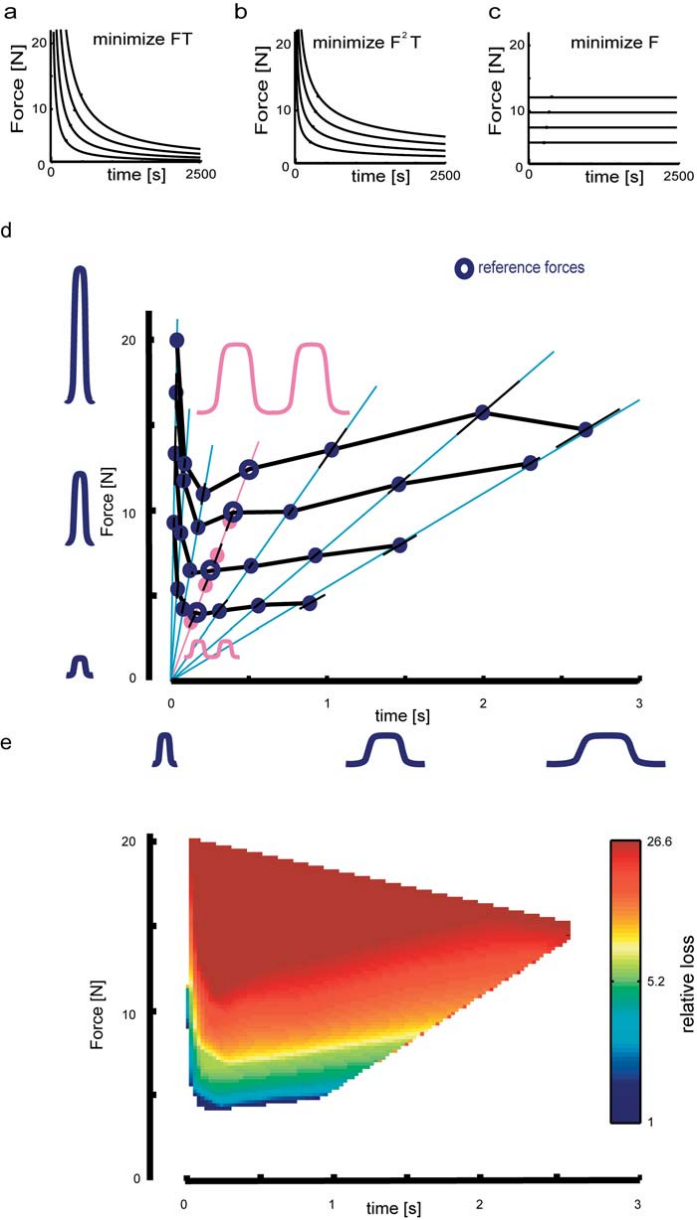
Hypothesized and Measured Indifference Curves and Loss Function from a Single Subject (A–C) The predicted indifference lines are shown that minimize (A) the integrated force *(F × T),* (B) the integrated squared force *(F^2^ × T),* and (C) the maximal force *(F)*. (D) Experimental data from a single subject. The open circles are the reference forces. The blue full circles connected by the black lines represent indifference points. Error bars denote the 95% confidence intervals. Force profiles are illustrated (blue curves for single forces, pink curve for doubling points). (E) Inferred color plot of the loss function (warmer colors represent greater cost).

Various hypothesized utility functions predict different choices and thus different indifference lines. The first model we hypothesized was that subjects would minimize the integrated force they are using *(F × T).* This predicts hyperbolas as indifference lines (Figure 2A). Alternatively, people could minimize the integrated squared force *(F^2^ × T)* (Figure 2B). In this case they would prefer long-duration, weak forces to short-duration, strong forces of equal integrated force. Another possible model would be that people would just try to minimize the maximal force they had to produce, regardless of how long they had to hold it *(F)* (Figure 2C).

A single subject's results are shown in Figure 2D. The reference forces are shown as open circles, while the indifference points are shown as filled circles. The location of the indifference points had relatively small error bars (black 95% confidence intervals). Therefore, this subject showed a preference for points towards the origin compared to those further from the origin (along the blue lines). Joining up such points in force–time space allows us to obtain indifference curves (black lines). For short duration profiles (less than 150 ms), as the duration increased, the force needed to decrease to maintain constant utility. This makes intuitive sense: as the duration of experienced force increases, more effort is required to stabilize the arm. For longer durations (greater than 500 ms), as the duration increased, the force required to maintain equal utility also increased. This means people prefer to experience a 2-s force profile compared to experiencing a 1-s force profile. We explain this counterintuitive result—that increasing both the duration and force can keep the utility constant—in the following way. The shape of the force profiles for all conditions was kept self-similar. This means that force profiles with a longer duration have a slow onset and offset (each is 20% of the total duration). For long durations subjects can, therefore, progressively compensate for the imposed forces as they ramp up slowly, thereby producing less loss.

We furthermore measured how much smaller a force profile needed to be (scaled uniformly in duration and force) so that experiencing it twice had the same utility as experiencing the unscaled profile once. For the four open-circle reference points in Figure 2D, the pink circles show the corresponding four points that have half the utility. We can thus infer how the loss function changes as the force profiles are scaled (Figure 2E; see Materials and Methods). Any order-preserving transformation of the utility function will have no effect on subjects' preferences. That means that arbitrary scalings can be applied to the loss function while the optimal behavior remains unchanged. This property of utility function is well known in economics and has led to the idea of ordinal utility (Pareto 1909), in which the ordering of preferences is the key feature of utility. The utility of the first reference point is thus arbitrarily set to be equal to one. The plotted relative utility is the utility function arising from this assumption. The double-hump experiment defines the derivative of the utility, which is interpolated and integrated to obtain the relative utility. To infer the relative utility function (Figure 2B), we had to assume local linearity. The loss function shows nonlinear behavior.

**Figure 3:**
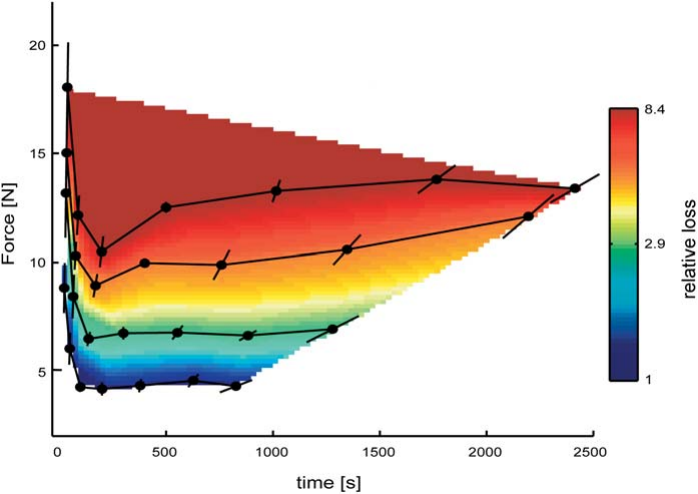
Iso-Loss Contours and Loss Function for the Set of All Subjects The black curves are the iso-loss curves. Error bars denote the standard error of the mean over the population. The color plot represents the inferred loss function (warmer colors represent greater loss) obtained by interpolating the data from the double-hump forces.

Figure 3 shows the inferred utility function averaged over all the subjects. We can analyze how loss increases along the line connecting the reference points (*F/T* = 44.6). Fitting a model of the form Loss = (*FT*)^α^ to the data from the double-hump experiments leads to an α of 1.1 ± 0.15 (mean ± SEM over subjects). This α, when fit to the data from all the subjects for each of the four lines, is approximately constant (1.2, 1.0, 0.9, 0.9). The shape of the loss function is highly conserved over the set of subjects. In particular, the effect that indifference curves increase for both very short and long durations is found over the set of subjects.

By applying the methodology developed by economists, we have shown that fundamental properties of the nervous system, such as loss functions, can be inferred by the choices humans make in a sensorimotor task. In general, these loss functions will depend on a large number of factors that were not measured in our experiment. For example, there are subjective emotional components to human decision making (Sanfey et al. 2003). However, parametric variations would allow such multi-dimensional loss functions to be determined. Interestingly, the inferred loss function we report cannot easily be modeled by any simple function of our experimental variables *F* and *T.* However, it is highly conserved across the subjects, suggesting a common underlying mechanism is at work. Moreover, our results suggest that the opposite approach—first hypothesizing a loss function and then predicting human decision making—is likely to miss interesting aspects of the behavior and underlying processes. We are therefore hopeful that the application of economic methods to the study of the nervous system, referred to as neuroeconomics (Glimcher 2003), will continue to provide new insights into the functioning of the central nervous system.

## Materials and Methods

### 

#### Subjects and the manipulandum

After providing written informed consent, five right-handed subjects (aged 20–40 y) participated in this study. The experiments were carried out in accordance with institutional guidelines. A local ethics committee approved the experimental protocols.

While seated, subjects held the handle of robotic manipulandum with two degrees of planar freedom. This was a custom-built device (vBot) consisting of a parallelogram constructed mainly from carbon fiber tubes that were driven by rare earth motors via low-friction timing belts. High-resolution incremental encoders were attached to the drive motors to permit accurate computation of the robot's position. Care was taken with the design to ensure it was capable of exerting large end-point forces while still exhibiting high stiffness, low friction, and also low inertia.

The robot's motors were run from a pair of switching torque control amplifiers that were interfaced, along with the encoders, to a multifunctional I/O card on a PC using some simple logic to implement safety features. Software control of the robot was achieved by means of a control loop running at 1,000 Hz, in which position and force were measured and desired output force was set.

A virtual reality system was used that prevented subjects seeing their hand, and allowed us to present visual images into the plane of the movement (for full details of the setup see Goodbody and Wolpert 1998) (see Figure 1A). The force between the subject's hand and the manipulandum was continuously measured using a six-axis force transducer (Nano25; ATI Industrial Automation, Apex, North Carolina, United States) sampled at 1,000 Hz by the control loop.

The experiment consisted of trials in which the robot generated force profiles on the subjects' hands. The force profiles experienced were parameterized by their duration *T* in ms and their maximal strength *F* in Newtons. The force profile *f* (*t*) approximated a square profile, but with smooth onset and offset:



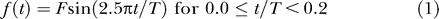





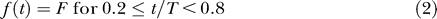





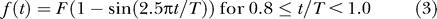



On each trial the subjects experienced two different force profiles and then could choose which of the two profiles to experience for a second time. Using such a forced-choice procedure allowed us to determine the indifference curves.

#### Inferring indifference pairs

Subjects saw a starting sphere and two selection spheres (see Figure 1A). Each trial started when the subject moved the cursor, representing their hand, into the starting sphere. The trial then had three phases. (1) One of the selection spheres turned green, and subjects were required to place the cursor into this sphere, where they experienced a force profile *F_1_*. The subjects then returned the cursor to the starting sphere. (2) The other selection sphere turned green, and subjects were required to place the cursor in that sphere, where they experienced a force profile *F_2_*. Subjects then returned the cursor to the starting sphere. (3) Both selection spheres turned green, and subjects were required to choose which of the two spheres to move to, where they would experience the same force associated with that sphere, either *F_1_* or *F_2_*. Therefore, subjects could decide which force profile, *F_1_* or *F_2_,* to experience a second time.

To obtain four indifference curves, we chose four reference profiles that had durations *T* of 200, 300, 400 and 500 ms. The maximal force *F* was chosen for each reference so that the ratio *T/F* had the value 44.6. This gave a maximal force that ranged from 4.5 N for the shortest duration reference to 11.2 N for the longest duration reference. These reference points lie along a straight line in time–force space (see Figure 2A, open circles).

On each trial, one of the two force profiles, *F_1_* or *F_2_,* was set to be one of the reference forces and the other was a test force. The sphere associated with the reference force was randomized each trial between the left and right locations. To obtain indifference lines, we wished to find points along the radial lines shown in Figure 2A to which subjects were indifferent to the four reference points. To obtain these we used a two-alternative forced-choice paradigm in which the test force produced was chosen from one of these lines, which correspond to *T/F* ratios of 2.0, 7.4, 20.0, 44.6 (double-hump, see below), 85.4, 142.1, and 203.0, with the aim of finding the point along the line at which subjects would choose between the reference and test force indifferently (that is, at probability level 0.5). We used an adaptive fitting protocol (QUEST; Watson and Pelli 1983) to find the *p* = 0.5 threshold of a logistic function. The reference points and *T/F* ratio lines were interleaved in a pseudorandom order. Forty trials were performed to obtain each indifference pair. Each reference point, together with the six *T/F* ratio line points that subjects preferred equally, defines an indifference curve.

#### Inferring the loss function

The above procedure allowed us to obtain indifference lines—where the utility has equal value. However, to obtain a full utility function we need to join up these lines and determine the relative utility of one indifference line to another. To achieve this we performed a two-alternative forced-choice paradigm in which the reference force was as before, but the test force was selected from the *T/F* = 44.6 line, with the force profile presented twice in succession (the “double hump” force). This condition was run interleaved with the other conditions. We assumed that the utility of experiencing the double hump was twice the utility of a single hump (a linearity assumption). This assumption allowed us to link the reference point to a point of half its utility, further allowing us to linearly interpolate log(utility) between these points to obtain estimates of the loss function between the lines.
